# Ant Colony Optimization Algorithm for Continuous Domains Based on Position Distribution Model of Ant Colony Foraging

**DOI:** 10.1155/2014/428539

**Published:** 2014-05-11

**Authors:** Liqiang Liu, Yuntao Dai, Jinyu Gao

**Affiliations:** ^1^College of Automation, Harbin Engineering University, 145 Nantong Street, Heilongjiang 150001, China; ^2^College of Science, Harbin Engineering University, 145 Nantong Street, Heilongjiang 150001, China

## Abstract

Ant colony optimization algorithm for continuous domains is a major research direction for ant colony optimization algorithm. In this paper, we propose a distribution model of ant colony foraging, through analysis of the relationship between the position distribution and food source in the process of ant colony foraging. We design a continuous domain optimization algorithm based on the model and give the form of solution for the algorithm, the distribution model of pheromone, the update rules of ant colony position, and the processing method of constraint condition. Algorithm performance against a set of test trials was unconstrained optimization test functions and a set of optimization test functions, and test results of other algorithms are compared and analyzed to verify the correctness and effectiveness of the proposed algorithm.

## 1. Introduction

Optimization is a kind of application technology using mathematical method to study how to search for the optimal solution for the problem in numerous solutions, as an important branch of science, which has been a widespread concern, and the rapid popularization and application in industrial production, economic and other fields. In the 1940s, with the increasingly widespread application of high-speed digital computers, optimization theory and algorithms developed rapidly and formed a new discipline. In recent years, swarm intelligence optimization theory has gradually developed into a new research direction of optimization techniques, typical algorithms with genetic algorithm [1], particle swarm optimization [2], ant colony optimization algorithm [3], artificial bee colony algorithm [4], firefly algorithm [5], and bat algorithm [6].

Inspired by the real ant colony foraging behavior in nature, early in the 1990s, the Italian scholars Dorigo et al. proposed ant colony optimization algorithm [3]. The algorithm adopts the distributed control, self-organizing, and positive feedback, and the optimization process does not depend on rigorous mathematical properties of optimization problem in itself and has the potential parallelism. Research on ant colony algorithm has shown that superiority of the algorithm for solving complex optimization problems. Because the ant colony optimization algorithm is essentially a kind of discrete optimization ideas, so the study of the optimization algorithm is mainly aimed at the problems of discrete domain optimization. But in real life, there are many optimization problems that are usually expressed as optimization problems of continuous domains. Therefore, how essentially discrete ant colony optimization algorithm would be applied to solve the optimization problems of continuous domains has become a new direction for research on ant colony optimization algorithm. In recent years, the studies of ant colony optimization algorithm for continuous domains have obtained some achievements and many scholars have proposed a variety of ant colony optimization algorithms for continuous domain [7–17]. Bilchev and Parmee first proposed a continuous ant colony optimization algorithm CACO [7], the algorithm for solving problems using genetic algorithms for global search of the solution space firstly and then using the ant colony optimization algorithm for local optimization to all the results. Dréo and Siarry proposed continuous interactive ant colony optimization algorithm CIAC [8], the algorithm modify the way of pheromone update and rules of path-searching, and use two ways of pheromone communication to guide ant optimization. Monmarché et al. proposed API algorithm [9]; All the ants set out from the same starting point, and each ant uses a complementary strategy that carried out optimization independently. Socha and Dorigo, who proposed continuous domain ant colony optimization algorithm ACO_R_ [10], used a Gaussian kernel probability density function express as distribution model of pheromone and gave ACO_R_ metaheuristic framework.

This paper proposes position distribution model of ant colony foraging and designs ant colony optimization algorithm for continuous domains based on the model to solve the standard test functions to verify the correctness and effectiveness of the algorithm. This paper is organized as follows. The relationship between the position distribution and food source in the process of ant colony foraging is analyzed in Section 2, and the position distribution model of ant colony foraging is given. To solve the unconstrained optimization problems and constrained optimization problems for ant colony optimization algorithm of continuous domains is designed in Section 3. The algorithm performance test trials and comparative analysis are given in Section 4. The conclusion is given in Section 5.

## 2. Position Distribution Model of Ant Colony Foraging

In the process of real-world ant colony foraging, people find that ant colony have a built-in optimization capability: they always can find the shortest path from nest to food. By studying this phenomenon, people propose the ant colony optimization algorithm.

**Figure 1:**
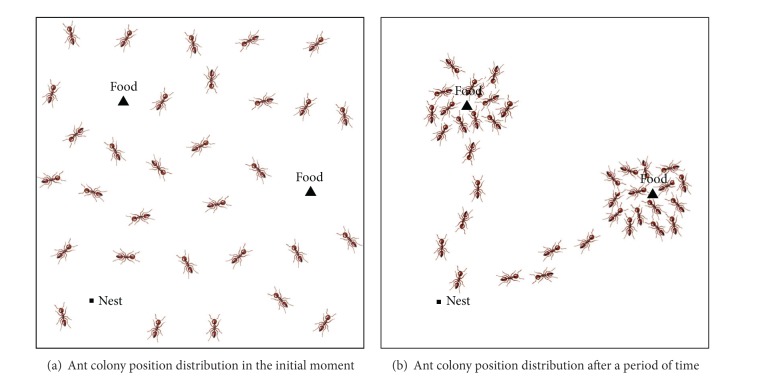
Process of ant colony foraging.

We can see the process of ant colony foraging from another perspective. As shown in Figure 1, an individual ant has no guidance of pheromone in the initial of foraging and searches for food sources blindly in the whole space; at this point, ant colony is distributed uniformly in the continuous space. As the process for feeding food, ants aggregated around the source will be increased, and the density of pheromone will increase in the vicinity, thus raising more ants to the food source. Also, the higher quality of the food source will attract a greater number of ants. Thus, in the process of ant colony foraging, the position distribution of ant colony and food source and quality is the same.

We can give such a model through the above process of analysis and expansion: assuming the food source is everywhere throughout in the continuous space, the quality of food source is different. At the initial moment, ants of ant colony distribute uniformly in the continuous space and release pheromones according to food sources of their position. The higher the quality of the food source, the more the pheromone ants released. The pheromone is distributed throughout the continuous space in a certain dispersed model, and ants perceive spatial concentration of pheromone intensity, moving to the position of a higher concentration of pheromone in a certain way and achieve the exploration of unknown regions during the move. The movement of the single ant will cause the change of the whole position distribution of ant colony, so that all the ants keep aggregating to the higher quality of food source and search the highest quality of food source in the continuous space eventually. This model is called position distribution model of ant colony foraging.

## 3. Ant Colony Algorithm of Continuous Domains Design

Below, we discuss the design process of ant colony optimization algorithm of continuous domain for solving unconstrained optimization problems and constrained optimization problems based on position distribution model of ant colony foraging.

### 3.1. Design of Algorithm for Solving Unconstrained Optimization Problem 

#### 3.1.1. Expression of Solution

Assuming the whole ant colony consists of *m* groups of substructure, each group contains *n* of ants. As shown in the following equation:

(1) $$\begin{array}{c} \left[\begin{array}{cccc} x_{1} & x_{2} & \cdots & x_{n}\\ \text{an}{\text{t}}_{11} & \text{an}{\text{t}}_{12} & \cdots & \text{an}{\text{t}}_{1n}\\ \text{an}{\text{t}}_{21} & \text{an}{\text{t}}_{22} & \cdots & \text{an}{\text{t}}_{2n}\\ \vdots & \vdots & & \vdots \\ \text{an}{\text{t}}_{m1} & \text{an}{\text{t}}_{m2} & \cdots & \text{an}{\text{t}}_{mn} \end{array}\right], \end{array}$$

the position ant_*ij*_ corresponding to the value *x*_*j*_ of the variable for* j*-ant in any subcolony *i*, the subcolony *i* of all the ants in the sequence of {ant_*i*1_, ant_*i*2_, …, ant_*in*_} represents a solution of the optimization problem.

#### 3.1.2. Distribution Model of Pheromones

In the position distribution model of ant colony foraging, each ant releases pheromone according to the quality of a food source of their position; pheromones are dispersed in the entire space, with increasing distance of the source and the concentration decreasing. Therefore, we need to choose a probability density function as distribution model of ant pheromone in optimization algorithm of continuous domains. Gaussian function is a common probability density function; we assume ants of the ant colony release pheromone externally on the function. At this point, *j* ant in any subcolony ant *i* corresponding to pheromone distribution model *τ*_*ij*_(*x*) can be expressed as

(2) $$\begin{array}{c} \tau_{ij}\left(x\right)=\frac{1}{\sqrt{2\pi }\sigma_{j}}e^{-({\left(x-\mu_{ij}\;\right)}^{2}/2\sigma_{j}^{2})\;},\\ \sigma_{j}=\frac{\left(u_{j}-l_{j}\right)}{\psi \cdot \left(1+\ln\left(n\right)\right)}, \end{array}$$

where *μ*_*ij*_ is the position ant_*ij*_ of ant *j* in the subcolony of ants *i*, namely, the distribution center, *σ*_*j*_  (*σ*_*j*_ > 0) means the width of the distribution function, *u*_*j*_ is the maximum allowable value of the variable *x*_*j*_, *l*_*j*_ is the minimum allowable value of the variable *x*_*j*_, *n* is the dimension of solution for the optimization problem, *ψ*  (*ψ* > 0) is a parameter, and *σ*_*j*_ is used to adjust size.

#### 3.1.3. Updating Position of Ant Colony

Before updating the position of ant colony, we need to choose a group as a parent from* m* subcolony. First, we use formula (3) to calculate each group of subcolony corresponding to the assessed value of solution. Consider the following:

(3) $$\begin{array}{c} \text{eva}{\text{l}}_{i}=\frac{1}{\left(1+e^{f(\text{an}{\text{t}}_{i1},\text{an}{\text{t}}_{i2},\ldots ,\text{an}{\text{t}}_{in})/T}\right)}, \end{array}$$

where *f*(ant_*i*1_, ant_*i*2_,…, ant_*i*n_) is the assessment value of the subcolony ant *i*; *T*  (*T* > 0) is the adjustment coefficient used to adjust the pressure of selection.

After assessment value for each group of subcolony is obtained, we calculate the selected probability for each group of subcolony according to

(4) $$\begin{array}{c} p_{i}=\frac{\text{eva}{\text{l}}_{i}}{\sum_{j=1}^{m}\text{eva}{\text{l}}_{j}}. \end{array}$$

Finally, we select parent colony *c* according to formula (5)

(5) $$\begin{array}{c} c=\{\begin{array}{cc} \arg\underset{i=1,2,\ldots ,m}{\max}\left(\text{eva}{\text{l}}_{i}\right), & q\leq q_{0},\\ C & q>q_{0}, \end{array} \end{array}$$

where *q*_0_  (0 ≤ *q*_0_ ≤ 1) is a given parameter, *q* is a random variable which distributed in [0,1] uniformly. *C* is a random variable which is generated according to formula (4).

After getting the parent ant colony* c*, the ant pheromone distribution model function *τ*_*cj*_(*x*) in the ant colony corresponding to random number generator for sampling, the *k* groups of children colony are generated. Then, according to the size of assessment value for each group of subcolony, we select the large assessment value of *m* group from (*m* + *k*) group of subcolony in order to achieve position of ant colony update.

### 3.2. Algorithm of Solving Constrained Optimization Problem

First, we define a solution *x* of measure constrained optimization problem violate measure for the degree of constraint condition:

(6) $$\begin{array}{c} \text{viol}\left(x\right)=\overset{r}{\underset{j=1}{\sum }}c_{j}\left(x\right). \end{array}$$

For inequality constraint *g*_*j*_(*x*) ≤ 0, *c*_*j*_(*x*) = max⁡{0, *g*_*j*_(*x*)}. For equality constraint *h*_*j*_(*x*) = 0,

(7) $$\begin{array}{c} c_{j}\left(x\right)=\{\begin{array}{cc} \left|h_{j}\left(x\right)\right|, & \left|h_{j}\left(x\right)\right|>h_{j\mathrm{Min}}\\ 0, & \left|h_{j}\left(x\right)\right|\leq h_{j\mathrm{Min}}, \end{array} \end{array}$$

where *h*_*jMin*⁡_ is a small positive number. viol(*x*) is equal to zero represent *x* is feasible solution. viol(*x*) which is greater than 0 represent *x* is infeasible solution.

When using this algorithm for solving constrained optimization problems, we allow infeasible solutions with probability *pMax*⁡  (0 ≤ *pMax*⁡≤1) existing. Algorithm calculation process is consistent with Section 3.1, and we only adjust the update process of the position of ant colony to the following process.

1. Calculate the number *e*Num = (*m* + *k*) × *pMax*⁡ of the maximum expected infeasible solutions in the group (*m* + *k*) of subcolony.
2. Calculate the number *r*Num of real infeasible solutions based on the value viol(*x*) in the group (*m* + *k*) of ant colony.
3. If *r*Num is less than *e*Num, then reserve the maximum of the assessment for group *m* of ant colony directly. If *r*Num is greater than *e*Num, the infeasible solutions are ranked by the value of viol(*x*), the greater number (*r*Num − *e*Num) of assessment value for the infeasible solutions from the value viol(*x*) is set to 0, and then reserve *m* group of the maximum fitness for (*m* + *k*) group of ant colony according to the assessment value.

## 4. Testing and Analysis of Algorithm Performance

### 4.1. Solution of Unconstrained Optimization Problems

In the process of solving unconstrained optimization problems algorithm performance testing, we refer to [10] method; the entire test is divided into three groups; the use of this algorithm with a kind of probabilistic learning methods, a kind of continuous domains ant colony algorithm, and a kind of metaheuristic methods is compared. The operating parameters of the algorithm design in this paper are shown in Table 1.

#### 4.1.1. Compare with a Kind of Learning of Probability Method

In this experiment, we use the algorithm in this paper to compare with [10, 21–24] which used a kind of probabilistic learning method for performance. In order to ensure the fairness of the results of comparison, the entire test method according to [10, 21] is given. The baseline function of this set of tests is given in Table 2. The stop condition of the algorithm is satisfied in

(8) $$\begin{array}{c} \left|f-f^{*}\right|<\varepsilon_{\min}, \end{array}$$

where *f* is the optimal solution for algorithm, f* is the known optimal solution, and *ε*_min⁡_ needs to satisfy the accuracy, taken as 10^−10^.

The comparative test results are shown in Table 3, where the results of other methods for solving are provided by [10, 21]. In the data of Table 3, the “1.0” represents the best algorithm for solving the extreme value of the basis functions. The actual median number of function evaluations is given in parentheses. Other algorithms corresponding evaluation data are the ratio of the evaluation number of the function and the best algorithms function when the stop condition is satisfied. “*∞*” represents the use of the algorithm that can not seek to satisfy the stop condition. The results marked “∗” represent the use of the algorithm to get the corresponding extreme value of the basis functions, not to satisfy stop condition results are found every time.

By the test results, it can be found that the algorithm has better searching capability and faster speed of convergence. In the process of solving seven of the basis functions, four functions of solution have results significantly better than other probability learning algorithms.

#### 4.1.2. Compare with a Kind of Ant Colony Algorithm of Continuous Domains

In this experiment, we use the algorithm in this paper and a kind of ant colony of continuous domains in [10] for performance comparison. The method of test is given according to [10]. The basis functions of this test are given in Table 4. The stop condition of algorithm is satisfied in

(9) $$\begin{array}{c} \left|f-f^{*}\right|<\varepsilon_{1}\cdot f+\varepsilon_{2}, \end{array}$$

where *ε*_1_ is relative error, the value is 10^−4^, *ε*_2_ is the absolute error, and the value is 10^−4^.

The results of comparative tests are shown in Table 5, where the percentage in brackets represents the minimum value of the independent use of the method for solving the corresponding basis functions 100 times, and ultimately the number of the stop conditions satisfied as a percentage of the total number of the algorithms is obtained. The symbol “—”represents that the algorithm is not used for solving the corresponding minimum of basis function; there is no data available for reference.

The results of this test prove that algorithm of this paper has better searching capability and faster speed of convergence. But we also find that the stability of the algorithm in this paper is relatively worse. In the process of solving the minimum of six basis functions, there are five solving functions which cannot guarantee that each stop solution condition satisfied the required accuracy.

#### 4.1.3. Compare with a Kind of Metaheuristic Method

In this experiment, we use the algorithm in this paper and a kind of metaheuristic method in [10] for performance comparison. The test is carried out according to methods given in [10]. The basis functions of this set of tests are shown in Table 6. In this experiment, except for the three basis functions given in Table 6, the function also uses* B*_2_ function, GP function, and* R*_2_ and* R*_5_ functions given in Table 4.

The results of comparative tests are shown in Table 7. We can find that algorithm of this paper has better searching capability and faster speed of convergence. But it also expose the instability of the algorithm.

When solving the minimum of the basis function Easom, stop condition to satisfy the accuracy requirements of the solution is found in the process of the algorithm independently running 100 times in only 43.

**Figure 2:**
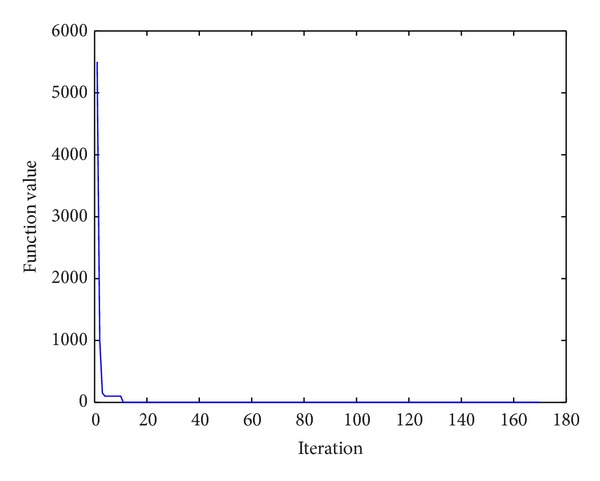
Curves of minimum function.

The algorithm for solving the convergence curve of the minimum of* B*_2_ function is shown in Figure 2. After the 158th iteration in the algorithm of this paper, the values of function have been less than 10^−10^; then the optimal solution has been found in the algorithm.

**Figure 3:**
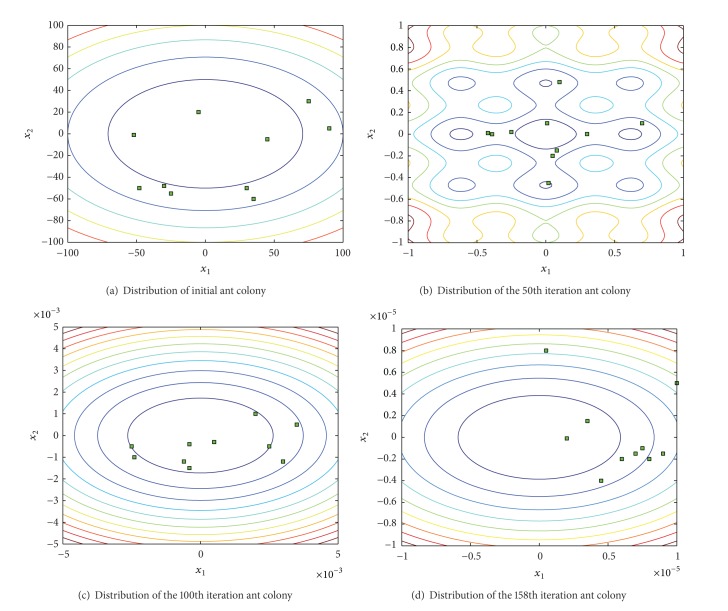
Change of the distribution of ant colony.

In the process of solving the function* B*_2_ in Figure 3, each group of ant colony corresponding to the solution with the change of the distribution of algorithm iteration is shown. Where the initial distribution of ant colony is shown in Figure 3(a), all initial ant colony corresponding positions are distributed in [−100,100]. With the operation of the algorithm, each group of ant colony rapidly approaches the optimal solution; in the 50th iteration, each ant of all the ant colony has been distributed in [−1, 1]. In the 158th step, the results of the algorithm for solving have satisfied stop conditions; then each ant colony is distributed in [−10^−5^, 10^−5^], and there are two groups of ant colony of overlapping position.

### 4.2. Solution of Constrained Optimization Problems

In this set of test experiments, we use this algorithm for solving the basis function G01~G12 of constrained conditions, and [18–20] are compared. In order to guarantee the fairness of the test results, the method of test is consistent with the methods of [18–20] adopted. Using the algorithm for solving various basis functions 50 times independently, best results are compared. In the process of running each algorithm, if the optimal value function obtained by consecutive 150 times does not change, the running algorithm exits. Otherwise, the algorithm exited after iteration 30,000 times.

During solving the basis test function of constrained conditions, the parameter values of the algorithm in this paper are shown in Table 8.

The results of comparative tests are shown in Table 9.

**Figure 4:**
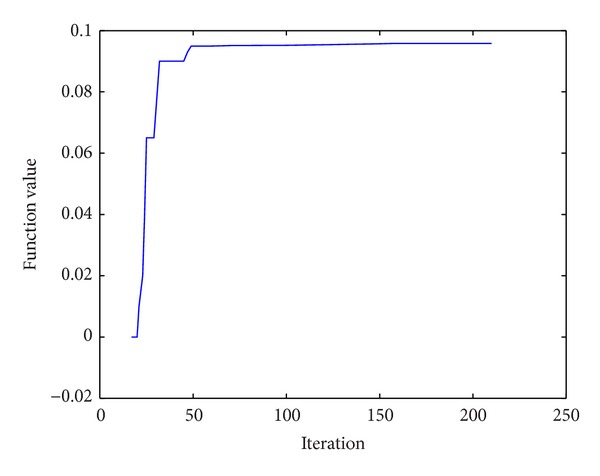
Maximum value curve of function.

We can see from the test results that effect of the algorithms in this paper for solving functions G01 and G02 was poor and solving the extreme values of function G03~G06, G08, G09, G11, and G12 gets minimum. It is evident that the algorithm in this paper for solving constrained optimization problems is effective. The algorithm in this paper for solving the maximum convergence curve of function G08 is shown in Figure 4.

In the process of solving, the feasible solution is found in the 17th iteration; after the 19th iteration, each group of subcolony corresponding solution is a feasible solution; the maximum value 0.095825 is founded by algorithm in the 157th iteration; all 10 groups of ant colony have found the maximum value in the 210th iteration.

**Figure 5:**
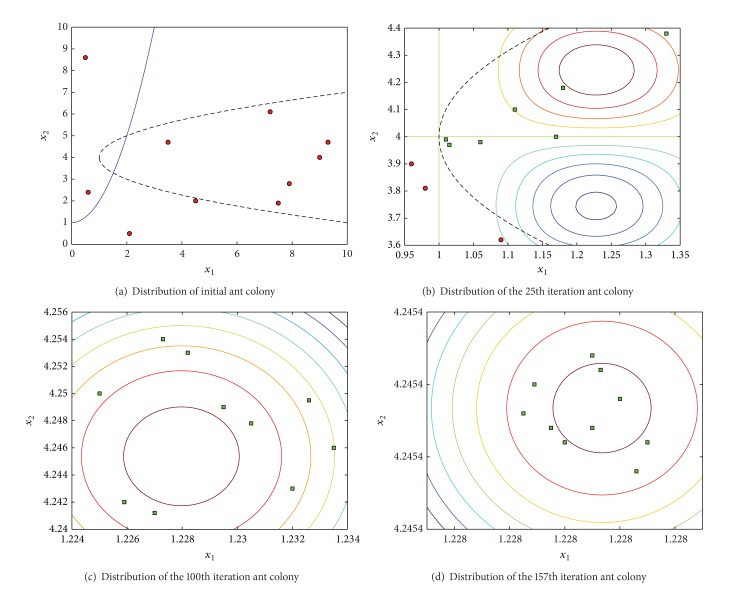
Distribution changes of ant colony.

The process of the algorithm in this paper for solving function G08 shown in Figure 5. Distribution changes of each group of ant colony corresponding to the solution with algorithm iteration are shown. The initial distribution of ant colony is shown in Figure 5(a); position of all ant colony does not satisfy all constraint conditions. There have been 7 groups of ant colony corresponding to the position that satisfied the constraint condition in the 25th iteration (Figure 5(b)); all 10 groups of ant colony corresponding to the position are distributed in *x*_1_ ∈ [1.227968,1.227973], *x*_2_ ∈ [4.245396,4.245377], and there is a group of ant colony that had found the optimal solution in the 157th iteration (Figure 5(d)).

## 5. Conclusion

In this paper, in the process of position distribution relationship between food sources of ant colony foraging for analysis, a new position distribution model by ant foraging is proposed. Any point in the solution space could be seen as a food source in the model, using multiple groups of subcolony for optimization; each group of subcolony represented a solution of the problem. In every iteration step, a group of ant colony was chosen from all subcolonies as the parent ant colony firstly and then sampled from pheromone density function of the group, generated children colony, and finally updated position of ant colony, so that each group of subcolony continued moving towards the solution space of the higher fitness value, converging to the optimal solution eventually. By simulating the above process, we designed ant colony optimization algorithm of continuous domains; a set of test functions for unconstrained optimization problems and a set of test functions optimization comparison test were compared and gave the solving process of the* B*_2_ test function and test function G08. Test results show that, in solving unconstrained optimization problems, the proposed algorithm has better searching capability and faster speed of convergence, but the stability of the algorithm is poor; when solving constrained optimization problems, the proposed algorithm has the basic optimization capability consistent with other algorithms.

## Figures and Tables

**Table 1:** Parameter values.

Parameter	*m*	*k*	*q*_0_	*ψ*
Values	100	50	0.9	4

**Table 2:** Basis functions of test 1 [10].

Function	Formula (*n* = 10)	Range	Optimum *f*(*x*)
Plane (PL)	$f_{\text{PL}}(\vec{x})=x_{1}$	$\vec{x}\in {\left[0.5,1.5\right]}^{n}$	1.5
Diagonal Plane (DP)	$f_{\text{DP}}(\vec{x})=\frac{1}{n}\overset{n}{\underset{i=1}{\sum }}x_{i}$	$\vec{x}\in {\left[0.5,1.5\right]}^{n}$	1.5
Sphere (SP)	$f_{\text{SP}}(\vec{x})=\overset{n}{\underset{i=1}{\sum }}x_{i}^{2}$	$\vec{x}\in {\left[-3,7\right]}^{n}$	0
Ellipsoid (EL)	$f_{\text{EL}}(\vec{x})=\overset{n}{\underset{i=1}{\sum }}{\left(100^{(i-1)/\left(n-1\right)}x_{i}\right)}^{2}$	$\vec{x}\in {\left[-3,7\right]}^{n}$	0
Cigar (CG)	$f_{\text{CG}}(\vec{x})=x_{1}^{2}+10^{4}\overset{n}{\underset{i=2}{\sum }}x_{i}^{2}$	$\vec{x}\in {\left[-3,7\right]}^{n}$	0
Tablet (TB)	$f_{\text{TB}}(\vec{x})=10^{4}x_{1}^{2}+\overset{n}{\underset{i=2}{\sum }}x_{i}^{2}$	$\vec{x}\in {\left[-3,7\right]}^{n}$	0
Rosenbrock (Rn)	$f_{\text{Rn}}(\vec{x})=\overset{n-1}{\underset{i=1}{\sum }}100{\left(x_{i}^{2}-x_{i+1}\right)}^{2}+{\left(x_{i}-1\right)}^{2}$	$\vec{x}\in {\left[-5,5\right]}^{n}$	0

**Table 3:** Results of test 1.

Function	This paper	(1 + 1) ES	CSA-ES	CMA-ES	IDEA
PL	**1.0** (15)	52.5	84	75.5	∞
DP	**1.0** (58)	14.4	21.7	18.8	∞
SP	**1.0** (199)	6.9	11	8.9	34.4
EL	3.2	66	110	**1.0** (4450)	1.6
CG	60.1	610	80	**1.0** (3840)	4.6
TB	**1.0** (550)	214.7	303.4	7.9	13.5
Rn	4.7*	51*	180	**1.0** (7190)	210*

**Table 4:** Basis functions of test 2 [10].

Function	Formula	Range	Optimum *f*(*x*)
*B*_2_	$f_{B_{2}}(\vec{x})=x_{1}^{2}+2x_{2}^{2}-0.3\cos\left(3\pi x_{1}\right)-0.4\cos\left(4\pi x_{2}\right)+0.7$	x→∈[-100,100]nn=2	0
Goldstein and Price	fGP(x→)=[1+(x1+x2+1)2(19-14x1+3x12-14x2+6x1x2+3x22)] ×[30+(2x1-3x2)2(18-32x1+12x12+48x2-36x1x2+27x22)]	x→∈[0.5,1.5]nn=2	3
Sphere model	$f_{\text{SM}}(\vec{x})=\overset{n}{\underset{i=1}{\sum }}x_{i}^{2}$	x→∈[-5.12,5.12]nn=6	0
Martin and Gaddy	$f_{\text{MG}}(x)={\left(x_{1}-x_{2}\right)}^{2}+{\left(\frac{x_{1}+x_{2}-10}{3}\right)}^{2}$	x→∈[-20,20]nn=2	0
Rosenbrock	$f_{\text{Rn}}(\vec{x})=\overset{n-1}{\underset{i=1}{\sum }}100{\left(x_{i}^{2}-x_{i+1}\right)}^{2}+{\left(x_{i}-1\right)}^{2}$	x→∈[-5,10]n *n* = 2.5	0

**Table 5:** Results of test 2.

Function	This paper	ACO_R_	CACO	API	CIAC
*R*_2_	**1.0** [95%] (62)	13.2	109.8	158.7	185.2
SM	**1.0** (69)	11.3	316.9	147.1	724.4
GP	**1.0** [97%] (54)	7.1	99.6	—	433.8 [56%]
MG	**1.0** [99%] (53)	6.5	32.5	—	221.3 [20%]
*B*_2_	**1.0** [95%] (80)	6.8	—	—	149.6
*R*_5_	1.4 [78%]	**1.0** [97%] (2487)	—	—	16 [90%]

**Table 6:** Basis functions of test 3 [10].

Function	Formula	Range	Optimum *f*(*x*)
Easom	$f_{\text{ES}}(\vec{x})=-\cos\left(x_{1}\right)\cos(x_{2})e^{-({\left(x_{1}-\pi \right)}^{2}+{\left(x_{2}-\pi \right)}^{2})}$	x→∈[-100,100]nn=2	−1
DeJong	$f_{\text{DJ}}(\vec{x})=x_{1}^{2}+x_{2}^{2}+x_{3}^{2}$	x→∈[-5.12,5.12]nn=3	0
Zakharov	$f_{\text{Zn}}(\vec{x})=\left(\overset{n}{\underset{i=1}{\sum }}x_{i}^{2}\right)+{\left(\overset{n}{\underset{i=1}{\sum }}\frac{ix_{i}}{2}\right)}^{2}+{\left(\overset{n}{\underset{i=1}{\sum }}\frac{ix_{i}}{2}\right)}^{4}$	x→∈[-5,10]nn=2.5	0

**Table 7:** Results of test 3.

Function	This paper	CGA	ECTS	ESA	DE
*B*_2_	**1.0** [95%] (80)	5.4	—	—	—
Easom	**1.0** (75) [43%]	19.6	—	—	—
GP	**1.0** [97%] (54)	7.7	4.3	14.5	—
*R*_2_	**1.0** [95%] (62)	15.5	7.7	13.2	10.1
*Z*_2_	**1.0** [98%] (48)	13	4.1	329.1	—
DJ	**1.0** (56)	13.3	—	—	7
*R*_5_	1.7 [78%]	1.9	**1.0** (2142)	2.5	—
*Z*_5_	**1.0** (81)	17	27.8	861.6	—

**Table 8:** Parameter value.

Parameter	*m*	*k*	*q*_0_	*ψ*	*p*Max
Value	100	50	0.1	4	0.2

**Table 9:** Results of test functions for solving constrained optimization problems [18–20].

Function	Known optimal	This paper	ES_SR_	KM	DP	PEPS_S
G01	−15.000	−13.934798	**−15.000**	−14.7864	**−15.000**	**−15.000**
G02	−0.803619	−0.781996	−0.803515	−0.79953	−0.803587	−0.803540
G03	−1.000	**−1.000**	**−1.000**	−0.9997	−0.583	**−1.000**
G04	−30665.539	**−30665.539**	**−30665.539**	−30664.5	−30365.488	−30665.538
G05	5126.498	5126.498	5126.497	—	—	5126.508
G06	−6961.814	**−6961.814**	**−6961.814**	−6952.1	−6911.247	**−6961.814**
G07	24.306	24.329	24.307	24.620	24.309	24.308
G08	−0.095825	**−0.095825**	**−0.095825**	**−0.095825**	**−0.095825**	**−0.095825**
G09	680.630	**680.630**	**680.630**	680.91	680.632	680.631
G10	7049.331	7078.146	7054.316	7147.9	—	7081.068
G11	0.750	**0.750**	**0.750**	**0.750**	**0.750**	**0.750**
G12	−1.000000	**−1.000000**	**−1.000000**	−0.999999857	**−1.000000**	**−1.000000**
